# Prevalence of Intolerance to Amines and Salicylates in Individuals with Atopic Dermatitis: A Systematic Review and Meta-Analysis

**DOI:** 10.3390/nu17101628

**Published:** 2025-05-09

**Authors:** Karen Fischer, Mark Jones, Hayley M. O’Neill

**Affiliations:** 1 Faculty of Health Sciences and Medicine, Bond University, Robina, QLD 4226, Australia; karen.fischer@student.bond.edu.au; 2 Institute for Evidence-Based Healthcare, Bond University, Robina, QLD 4226, Australia; majones@bond.edu.au

**Keywords:** atopic dermatitis, eczema, food intolerance, salicylate intolerance, acetylsalicylic acid, histamine intolerance, amine intolerance, elimination diet

## Abstract

Background/Objectives: Elimination diets targeting amines and salicylates have been used since the 1980s to diagnose pharmacological food intolerance in individuals with atopic dermatitis (eczema), yet supporting evidence regarding relevance is limited. To our knowledge, this systematic review with meta-analysis is the first to examine the prevalence and association between atopic dermatitis flares and amine intolerance (including histamine intolerance) and salicylate intolerance in individuals with atopic dermatitis. Methods: Following the PRISMA guidelines, searches of PubMed, Embase, CINAHL, and Cochrane were conducted. Included studies involved children and adults with atopic dermatitis who underwent dietary elimination and double-blind placebo-controlled challenges involving histamine, other amines, or salicylates. Risk of bias was assessed using the Joanna Briggs Institute Checklist for Prevalence Studies. Meta-analysis of the prevalence of atopic dermatitis flare by food chemical type was performed using a random effects model in Stata/MP, version 16.1 for Windows. Heterogeneity was assessed using the I^2^ statistic and Cochrane’s Q test. Results: A total of 2323 titles and abstracts were screened, with 46 full-text articles evaluated independently by two reviewers, resulting in the inclusion of four prevalence studies involving 188 participants. Meta-analysis of two trials showed histamine intolerance prevalence at 31% [95% CI, 20–41%] with no heterogeneity (I^2^ = 0.01%). Two trials showed amine intolerance prevalence at 32% [95% CI, 16–48%] with moderate heterogeneity (I^2^ = 34.91%). Two trials showed salicylate intolerance prevalence at 53% [95% CI, 44–62%] with no heterogeneity (I^2^ = 0.00%). Conclusions: This review suggests there may be low-certainty evidence linking intolerance to histamine, amines, and salicylates to atopic dermatitis flare in a substantial proportion of individuals with atopic dermatitis. Further, well-designed studies are needed to confirm these findings and clarify the role of pharmacological food intolerance in atopic dermatitis.

## 1. Introduction

Atopic dermatitis (AD) is a common inflammatory skin disease with a global prevalence of around 129 million [1]. Its clinical course of remission and flares involves symptoms such as erythematous rash, localised lesion weeping and oozing, and intense itch [2,3]. An AD flare is defined as worsening symptoms that may result in seeking medical advice or an escalation of treatment [4]. These flares may be linked to stress [5], infection [6] and environmental irritants and allergens [7], including food allergy [8,9], and food intolerance [10,11,12,13], which is a non-IgE-mediated adverse reaction to a substance consumed in a usually tolerable dose [14,15]. The preference of AD patients and caregivers for non-drug treatments [16] means dietary elimination is increasingly explored, with approximately 75% of AD patients omitting certain foods from their diet [17,18]. While the association between AD and food allergy is established [9,19] and can be measured through various immunoglobulin E (IgE) sensitisation tests [20], the possible link between AD and pharmacological food intolerance has limited representation in the literature. Diagnostic difficulties [21], such as the absence of validated biomarkers [22], as well as the high cost and logistical burden of blinded oral challenges hamper research in this area. In addition, symptom observation and dietary elimination protocols are time intensive and require sustained participant compliance [23]. Moreover, individual tolerance thresholds to these food chemicals vary widely [24], making it essential to administer safe, incremental dosages during oral challenges to ensure reliable and interpretable results.

Regardless, research targeting food intolerance, including histamine intolerance [15,22,25,26] (a type of amine intolerance) and salicylate intolerance [12,27], has been employed since the 1980s in individuals with AD [10,11,12,13]. Collectively known as pharmacological food intolerance (also referred to as pharmacologic [28]), this adverse food reaction subgroup is a non-immune-mediated, reproducible adverse reaction to naturally present or added food chemicals that can produce a drug-like effect [29,30]. Symptoms may mimic food allergy symptoms [28,29,30], including pruritus, flushing, swelling, tachycardia, diarrhoea, rhinitis, and palpitations [21,26]. Pharmacological food intolerance subgroups include amine intolerance [12,31] (group of various amines), histamine intolerance [10,11,31,32], salicylate intolerance [12,30], and intolerance to caffeine [30] and theobromine [30,33]. These pharmacological food chemicals are common in habitual Western diets [34,35] and medications [36,37,38]; however, their contribution to AD flare is unclear.

Biogenic amines, including histamine, phenylethylamine, and tyramine, are organic compounds formed by the decarboxylation of amino acids by microorganisms [39]. This process increases the amine content of foods, particularly when protein-rich foods, such as meat, cheese, and fish, are cooked and stored overnight. Moreover, fish spoilage, where elevated histamine reaches toxic levels, can result in food poisoning [40]. In contrast, amine intolerance, including histamine intolerance, involves reproducible adverse reactions to non-toxic levels of dietary amines in dosages that most individuals could safely consume. Dietary histamine and other amines are present at significant levels in cured or grated cheese, preserved or canned fish, eggplant, spinach, and fermented products including sauerkraut, soy, and sausages, and at lower levels in tomato, cured (deli) meats, beer, and wine [41]. Histamine-free foods that contain other amines include soy, egg, yoghurt, citrus fruit, alcohol, and banana [21], to name a few. Additionally, the histamine level in food naturally increases through storage, fermentation, and cooking methods, including frying and grilling, except for boiling [42]; thus, a diet may be low in histamine but is unlikely to be histamine-free. Histamine is not present in dairy products (unless fermented), eggs, fresh seafood, or meat when prepared and eaten fresh [41]; therefore, calcium and iron deficiencies are unlikely to be major nutritional concerns when following a low histamine diet.

Identifying histamine intolerance in an individual usually involves ruling out other causes (such as food allergy), symptom observation, and following a low histamine diet. This involves temporary dietary exclusion of histamine-rich foods for a minimum of one week (although histamine exclusion for three weeks has resulted in higher remission rates [21]). This is typically followed by oral challenge with encapsulated histamine hydrochloride at varying strengths [10,11] or open challenge [23] with histamine-rich food [32].

Salicylate intolerance symptoms include nasal polyps [43,44], erythematous and pruritic rash (often involving hands and feet) [45], abdominal pain [45], nausea [45], chronic rhinosinusitis [36,46], lower respiratory tract symptoms [45], and asthma [46]. Salicylates are a group of bioactive compounds found in acetylsalicylic acid medications [36,37], including aspirin and nonsteroidal anti-inflammatory drugs (NSAIDs), infant teething gel [47,48], and foods, including but not limited to, sweet potato (yam), cumin, cocoa, coffee, kiwifruit (kiwi), watermelon, grapes, apricot, coconut, pears (with skin on), and dates and other dried fruits [49,50]. While fruit restriction may lower an individual’s vitamin C and beta-carotene intake, unflavoured dairy products and fresh meats are naturally free of salicylates; therefore, calcium and iron deficiencies are unlikely to occur when following a well-planned low-salicylate diet. Since the 1980s, low salicylate diets have been explored [12,13] to determine salicylate intolerance in patients presenting with chronic disorders such as AD [12,13], asthma [51], and irritable bowel syndrome [12,52], among others. Salicylate intolerance is associated with AD-related atopic conditions, including asthma [27,46,51,53] and chronic rhinosinusitis [36,46], urticaria [51], NSAID-exacerbated respiratory disease (N-ERD), and aspirin-exacerbated respiratory disease (AERD) [54]. Yet, the prevalence and association between intolerance to salicylates and amines and AD flare is underexplored [12,13].

This systematic review aims to address this gap by critically appraising the literature regarding the prevalence of pharmacological food intolerance involving dietary histamine, amines, and salicylates in individuals with AD, examining the association between these food chemicals and AD flare.

## 2. Materials and Methods

### 2.1. Search Strategy

A preliminary search was conducted on PubMed and Google Scholar to identify relevant literature. The protocol was developed and registered in the International Prospective Register of Systematic Reviews (PROSPERO), registration number CRD42023432950. This review followed the 2020 Preferred Reporting Items for Systematic Reviews and Meta-Analysis (PRISMA) guideline [55]. Ethics approval was not required as this study is a systematic review involving published data.

Systematic searches of PubMed, Embase via Elsevier, Cochrane Library for trials in Cochrane Central Register of Controlled Trials (CENTRAL), and CINAHL via Ebsco were conducted on 20 April 2023. Grey literature searches of ProQuest and Google (incognito window) were performed between 20 April 2023 and 27 April 2023. PubMed, Embase, and CINAHL searches were repeated on 22 April 2024 with date restrictions, yielding 169 results and no new included studies (eMethods in the Supplement).

The search was designed with the assistance of a health science librarian. We did not apply exclusion criteria based on publication type or language. Non-English papers were translated via Google Translate. Forward and backward citation searches involving eligible studies were conducted. This research was supported by an Australian Government Research Training Program Scholarship.

### 2.2. Inclusion and Exclusion Criteria

All trial types were eligible, including randomised controlled trials (RCTs), non-randomised trials, and single-arm pre–post interventions. Case studies were excluded. We included studies involving adults and children of all ages with AD, including search terms such as eczema, neurodermatitis, and Besnier’s prurigo. Studies that included participants with other disease states were reviewed, but only data on participants with AD were extracted and analysed. Eligible interventions involved dietary elimination (alone) or elimination followed by the reintroduction of foods via double-blind placebo-controlled challenges (DBPCC) [23,56]. Our target pharmacological food intolerance chemical subgroups were salicylates, including acetylsalicylic acid, salicylic acid, willow bark and aspirin; amines, including tyramine, tryptamine, phenylethylamine, and histamine; caffeine, including coffee; and theobromine, including chocolate. 

We excluded studies reporting only on immune-mediated food allergies, pregnancy and breastfeeding, contact dermatitis, dermatitis herpetiformis, seborrheic dermatitis, environmental allergens, or dietary supplements, as well as non-human studies. Due to the nature of prevalence studies, participants could be their control, or the study could involve a control group.

Our primary outcome was identifying the prevalence of intolerance to dietary amines (including histamine as a separate group), salicylates, caffeine, or theobromine in individuals with AD. The intolerance reaction of interest was a significant exacerbation (flare) of AD, whether acute and/or delayed, after oral challenge involving our targeted food chemical subgroups, as measured by a validated tool, such as SCORing Atopic Dermatitis (SCORAD) [57], or a non-validated scoring method. Our secondary outcome was clinically significant improvement of AD symptoms during dietary elimination, as measured by a validated tool or a non-validated scoring method. The third outcome was other adverse reactions from oral challenge (e.g., urticaria). All study settings were eligible, including hospitals, dermatology clinics, and allergy clinics.

Search results were imported into EndNote^TM^ 21 for deduplication and then into Covidence^TM^ for screening. A total of 2323 titles and abstracts were screened independently by two researchers using predefined selection criteria. Full-text screening of 46 eligible studies was conducted independently by two researchers. Disagreements were resolved by discussion. Study quality and risk of bias were assessed independently by two researchers using the Joanna Briggs Institute (JBI) Checklist for Prevalence Studies [58] and visualised by Risk-of-bias VISualization [59] (Table S1 and Figure S1 in the Supplement). Disagreements were resolved by discussion. The GRADE Handbook [60] by the Grading of Recommendations Assessment, Development and Evaluation (GRADE) Working Group was consulted for the quality of evidence.

### 2.3. Data Extraction

Data extraction was conducted independently by two researchers, and disagreements were resolved by discussion. The following data were extracted from each included study: 1. study characteristics (e.g., author, publication year, country, study design); 2. participant characteristics (e.g., age, number of males, AD severity, sample size); 3. chemical subgroup (e.g., amines), dose, oral challenge responders, diet responders, scoring tool (Table 1), and other adverse reactions from oral challenge.

### 2.4. Data Analysis

We used a random effects model in Stata/MP, version 16.1 for Windows, to estimate the average proportion of people with AD having pharmacological food intolerance. Inconsistencies between the studies were assessed using the I^2^ statistic and Cochrane’s Q test, with heterogeneity considered high if the I^2^ statistic was >50%. Given the expected heterogeneity due to differences in food chemical type and methods to test pharmacological food intolerance, the prevalence of AD flare was meta-analysed by food chemical type (e.g., histamine). Subgroup analysis was planned for age groups (adults versus children) and AD severity (mild, moderate, and severe).

## 3. Results

**Figure 1:**
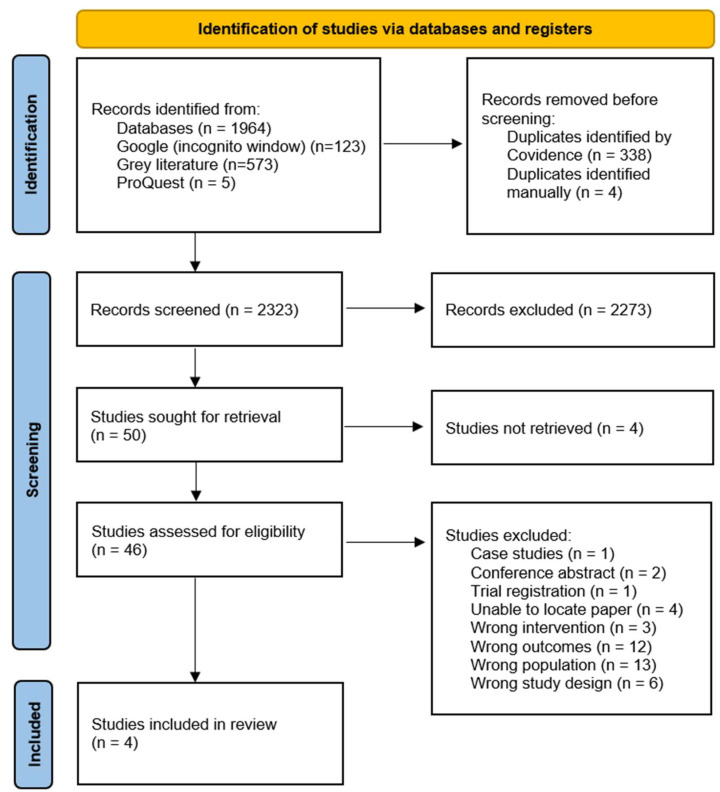
PRISMA flowchart of the study selection process.

Figure 1 illustrates the study selection process. The search identified 2323 non-duplicate records. Of these, 46 studies were eligible for full-text screening. The JBI Checklist for Prevalence Studies [58] identified five studies [32,61,62,63,64] with missing data and high risk of bias; therefore, these were excluded in accordance with the Cochrane Handbook for Systematic Reviews of Interventions [65]. We initially aimed to investigate a range of pharmacologically active food chemicals including caffeine and theobromine, using search terms such as coffee and chocolate. However, several studies were ultimately excluded due to poor reporting quality [63,64,66,67,68,69]. For example, Čelakovská et al. [63] collected retrospective self-reports of hypersensitivity reactions to foods, including chocolate and cocoa, in patients aged 14 years and older with AD. However, this study did not specify the type of adverse reaction, making it unclear whether the reported hypersensitivity reactions aggravated AD or triggered other conditions. Additionally, the use of self-reported data introduced a high risk of recall bias, subjectivity, and inaccuracy. The absence of external validation further contributed to a critical risk of bias. A study by Maintz and colleagues [25] was excluded as a subgroup of participants with AD and suspected histamine intolerance were placed on a low histamine diet; however, the wrong outcomes were reported. Five studies [66,67,68,69,70] reporting on chocolate intolerance were excluded due to poor reporting, missing data, and critical risk of bias. Moreover, chocolate contains multiple pharmacologically active chemicals, including theobromine [71], caffeine [72], salicylates [49], histamine [73], and other amines [73], and typically includes dairy and soy, which are known to exacerbate AD [9]. As a result, the specific compound(s) responsible for adverse reactions to chocolate remain unclear and may not be attributed solely to pharmacological food intolerance. However, for transparency, the characteristics of excluded studies investigating chocolate intolerance are presented in the Supplement (Table S2 in Supplement), along with coffee and caffeine intolerance (Table S3 in Supplement).

Ultimately, four prevalence studies [10,11,12,13] with 188 participants were included in the systematic review and meta-analysis. All included studies [10,11,12,13] involved double-blind placebo-controlled challenges (DBPCC) [23,56] to determine prevalence. DBPCC is considered the criterion standard [74] and gold standard method of food intolerance and food allergy diagnosis [23,56,75]; hence, it is a reliable and suitable test method. Subgroup analysis for age groups and severity of AD was not possible due to missing data.

### 3.1. Histamine Intolerance

**Figure 2:**
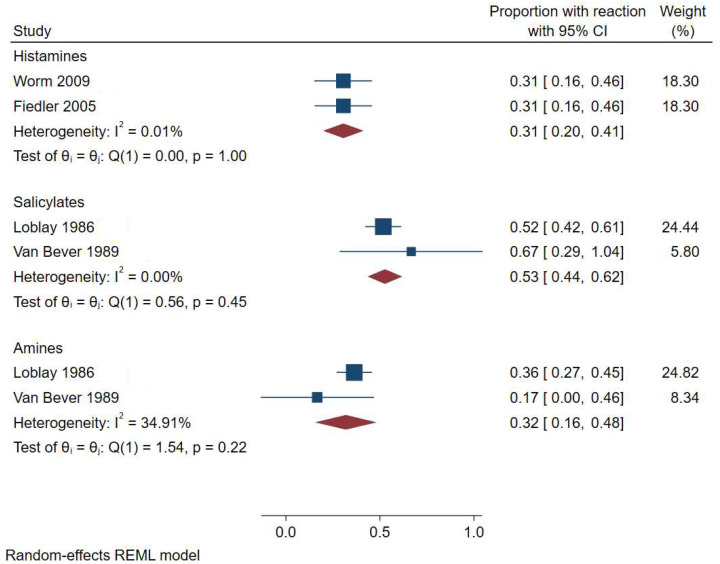
Meta-analysis [10,11,12,13] of the proportion of participants with atopic dermatitis flare after food challenge stratified by food chemical.

Two pre–post interventional studies [10,11] investigated dietary elimination of histamine, followed by DBPCC using histamine hydrochloride administered in capsules in two dosages of increasing strength to determine histamine intolerance. Meta-analysis (Figure 2) showed the prevalence of histamine intolerance across two trials was 31% [95% CI, 20–41%], with no heterogeneity (I^2^ = 0.01%) [10,11].

**Figure 3:**
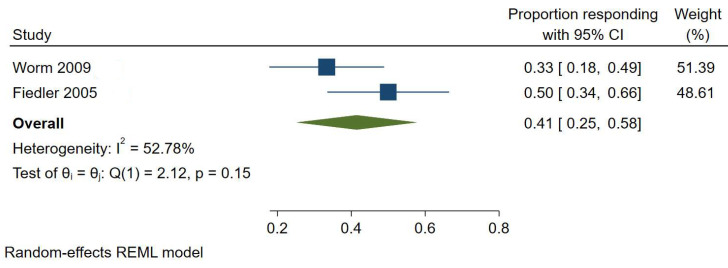
Meta-analysis [10,11] of the proportion of elimination diet responders in studies involving a low histamine diet.

There were only sufficient studies for meta-analysis of the proportion of diet responders in the histamine intolerance studies. Meta-analysis of two trials [10,11] showed low-certainty evidence that histamine-restricted diets may reduce AD severity in an average of 41% of participants [95% CI, 25–58%] (Figure 3). There was evidence of high heterogeneity (I^2^ = 52.78%), reflecting variation in the proportion of participants responding to a low histamine diet across included studies—ranging from 25% to 58% of participants recording significant improvement in AD severity after one week on a low histamine diet.

### 3.2. Amine Intolerance

Two single-arm, pre–post interventional studies [12,13] investigated amine intolerance using an elimination diet followed by DBPCC. Meta-analysis of these studies estimated a pooled prevalence of 32% [95% CI, 16–48%], with moderate heterogeneity detected (I^2^ = 34.91%) (Figure 2).

### 3.3. Salicylate Intolerance

Two single-arm, pre–post interventional studies [12,13] investigated salicylate intolerance using an elimination diet followed by DBPCC. The meta-analysis of these trials yielded a pooled a prevalence of 53% [95% CI, 44–62%] for salicylate intolerance, with no observed heterogeneity (I^2^ = 0.00%).

### 3.4. Other Adverse Reactions from Oral Challenge

Aside from AD flare, other adverse symptoms from oral challenge involving encapsulated chemical(s) were reported in the included studies. Oral histamine challenge elicited allergy-like symptoms, including flushing [10,11], headache [10,11], vertigo [10], hypotension [10], nausea [11], tachycardia [11], and dizziness [11]. Van Bever et al. [13] reported that amine challenge involving tyramine caused abdominal pain with diarrhoea. Loblay and Swain [12] reported that oral challenge with acetylsalicylic acid caused urticaria, gastrointestinal symptoms, headache, and cerebral symptoms in participants with AD, suggesting that amine and salicylate intolerance may be associated with other adverse events. Therefore, clinical symptoms of pharmacological food intolerance may mimic those of food allergies [76], suggesting its potential role as a differential diagnosis when a food allergy is suspected but not confirmed by validated allergy tests [77].

### 3.5. Risk of Bias

Using the JBI Checklist for Prevalence Studies [58], two trials [10,13] were rated as having a low risk of bias, and two trials [11,12] as having a high risk (Figure S1 in the Supplement). Reasons for high risk of bias included study limitations involving missing data (older studies were generally poorly reported) and failure to measure outcomes in a standardised and reliable way. Van Bever et al. [13] administered both an elemental diet and double-blind placebo-controlled challenges involving tyramine via nasogastric tube in a hospital setting, which contributed to a low-risk-of-bias rating. Although the GRADE Handbook [60] was consulted for quality of evidence assessment, its lack of guidance on prevalence studies prevented full implementation; however, relevant questions were addressed (Table S4 in the Supplement).

## 4. Discussion

### 4.1. Main Findings and Their Significance

To our knowledge, this systematic review with meta-analysis is the first to critically examine the association between AD flares and intolerance to histamine, amines, and salicylates. Overall, the findings suggest that a subset of individuals with AD may experience symptom exacerbation in response to these pharmacological food compounds. The outcomes are summarised in Table 2.

Histamine intolerance was evaluated in two studies (n = 72) using a low histamine diet, with an average of 41% of participants showing clinically significant improvement after one week on a low-histamine diet [10,11]. Subsequent oral challenge with histamine hydrochloride confirmed histamine intolerance in 31% of participants across the two studies [10,11]. Amine intolerance, evaluated in two studies using oral challenge with tyramine and phenylethylamine, was identified in 32% of participants [12,13]. The meta-analysis revealed a similar overall prevalence to histamine intolerance. However, moderate heterogeneity (I^2^ = 34.91%) indicates variability, which may indicate differences in challenge protocols, population characteristics, and outcome assessments.

Salicylate intolerance demonstrated the highest pooled prevalence at 53%, with no observed heterogeneity between studies. While synthetic salicylates (e.g., acetylsalicylic acid) were used in challenges rather than food-based sources, the consistent response across trials supports the need for further research using dietary salicylates.

### 4.2. Potential Mechanisms for a Link Between Amine and Salicylate Intolerance and AD

Histamine intolerance is thought to result from impaired degradation of histamine in the body due to reduced activity of enzymes diamine oxidase (DAO) and histamine-N-methyltransferase (HNMT) [21]. DAO deficiency may be genetic, caused by certain pharmaceutical drugs, or caused by unknown factors, leading to elevated circulating histamine levels [21,78]. Gut dysbiosis, with an overgrowth of histamine-producing bacteria, may also contribute to histamine intolerance [79]. Serum DAO, DAO-encoding gene variants, and metabolic urinary markers are being investigated as potential biomarkers for histamine intolerance [21,78]. Notably, one study [25] observed significantly lower serum DAO levels in patients with AD and histamine intolerance compared to healthy controls, supporting a potential mechanistic link between histamine intolerance and AD exacerbations.

Salicylate intolerance is a non-immune-mediated response that is associated with cyclooxygenase-1 (COX-1) and cyclooxygenase-2 (COX-2) enzyme inhibition [80,81]. Acetylsalicylic ingestion irreversibly blocks COX-1, which amplifies inflammatory responses in salicylate-intolerant individuals, causing a significant decline in prostaglandin E2 (PGE2) production (a key inflammatory mediator) [82]. PGE2 suppression is not seen in healthy individuals [83]. In salicylate-intolerant individuals, reduced PGE2 leads to an overproduction of cysteinyl leukotrienes (CysLTs), which upregulates interleukin-33 (IL-33) [83], leading to Th2-mediated inflammatory and allergic responses [51], bronchoconstriction, and, possibly, AD symptomology [84,85]. IL-33 is an inflammatory cytokine, which is commonly elevated in individuals with AD [86], implicated in itch and disruption of the skin barrier [87], and linked to salicylate-intolerant individuals [83], providing a further potential link. Elevated CysLTs are commonly found in patients with NSAID-exacerbated respiratory disease (N-ERD) and aspirin-exacerbated respiratory disease (AERD) [82,83], with urinary leukotriene E4 (uLTE_4_) being explored as a candidate biomarker to identify salicylate intolerance in these patients [82]. N-ERD and AERD are associated with atopic comorbidities, including asthma and chronic rhinosinusitis (also known as atopic march) [88], supporting the plausibility of a shared mechanism in AD, possibly through non-IgE-mediated pathways involving leukotriene overproduction. Further exploration of these pathways is warranted in future trails involving individuals with AD.

### 4.3. Clinical Implications

The European Guideline on Atopic Eczema (2022) [89] supports diagnostic elimination diets and challenge tests in patients with suspected food-triggered AD. While most current dietary investigations focus on IgE-mediated allergies to egg, dairy, wheat, and other common foods [9], our findings suggest that non-IgE-mediated food intolerances may also contribute to disease exacerbations. Notably, eggs, dairy, and wheat are free of histamine and salicylates [90], with the exception of fermented products like cheese and yoghurt, reducing the likelihood that typical elimination diets would address pharmacological food intolerance.

While salicylate intolerance in Europe is estimated at 1.9% [46], this meta-analysis found that 52% of individuals with AD may be intolerant to salicylates. Therefore, caution may be advised when prescribing dietary modifications, salicylate-based medications, and teething gel to this population. Furthermore, in comparison to the general prevalence of histamine intolerance, which is approximately 1–3% of the population [21,91], individuals with AD may be at higher risk of adverse events from exogenous histamine, although more research is needed before clinical recommendations can be made.

Caution is warranted in the interpretation and application of these findings, as restrictive diets can compromise micronutrient intake, particularly vitamin A (beta-carotene) and calcium [92]. These risks can be mitigated through dietitian supervision and the use of appropriate supplementation [93]. Still, elimination diets should be avoided in vulnerable individuals, including children with impaired growth, individuals with eating disorders or mental health conditions, and those who are underweight or frail. Given that pruritus is a primary driver of disease burden in AD [94] and that pharmacological food chemicals can exacerbate pruritus via non-IgE-mediated mechanisms [87], supervised dietary investigation targeting histamine, amines, and salicylates may offer clinical benefit in selected patients. Nevertheless, individualised assessment and structured challenge testing are essential to minimise unnecessary nutritional restrictions and ensure nutritional adequacy.

### 4.4. Strengths and Limitations

A major strength of this review is the inclusion of studies using DBPCC to confirm AD flare responses, providing more robust evidence than studies relying on self-reported outcomes. The inclusion of both adult and paediatric populations also enhances the generalisability of the findings. However, several major limitations should be noted. We were unable to fully assess the certainty of evidence using the GRADE guidelines, as they are not specifically designed for prevalence studies. The small sample sizes reduced statistical power, and two studies [12,13] employed non-validated symptom scoring tools, limiting the reliability of the outcome assessment. Inconsistent symptom measurement methods (e.g., SCORAD vs. unstructured physician assessment) further reduced comparability across studies. Poor reporting and missing data were also common, which is typically seen in older studies. We also could not perform subgroup analysis on age and severity reducing clinical applicability. Furthermore, the absence of randomised controlled trials (RCTs) limits the ability to draw conclusions about causality. Although RCTs involving elimination diets are challenging—due to difficulties with blinding, high costs, participant burden, and dropout risk—they are needed to strengthen the evidence base. Overall, the certainty of evidence linking pharmacological food intolerance to AD flares remains low and requires confirmation through well-designed, high-quality trials.

## 5. Conclusions

Low-quality evidence suggests that intolerance to histamine, amines (specifically, phenylethylamine and tyramine), and salicylates (acetylsalicylic acid) may play a role in triggering AD flares in a subset of individuals with AD. While elimination diets targeting these compounds may show potential for improving symptoms and quality of life, the current evidence remains insufficient for clinical recommendation. This systematic review highlights a critical gap in the understanding of pharmacological food intolerance in AD. Future high-quality RCTs, with adequate sample sizes, employing validated flare assessment tools and standardised oral challenge methods, including food-based challenges, are needed to confirm these preliminary associations. Given patients’ and caregivers’ preference for non-medical treatments for AD, it is time to revisit dietary strategies as potential non-drug interventions for managing this chronic and burdensome condition.

## Figures and Tables

**Table 1:** Characteristics of included studies.

Source	Study Design	Country	Intervention	*n*, Total	*n*, Male	Age, Mean (SD), Range	Scoring Method	Chemical, Dose	*n*, Oral Challenge Responders	*n*, Diet Responders
Fiedler et al. [11]; 2005	Single-arm pre–post intervention	Germany	Elimination diet then DBPCC	36	NR	NR	SCORAD	Histamine hydrochloride, 0.75 mg/kg and 1.5 mg/kg body weight	11	18
Loblay et al. [12]; 1986	Single-arm pre–post intervention	Australia	Elimination diet then DBPCC	110	40	NR	Physician assessment	Acetylsalicylic acid, 300 mg Phenylethyl-amine, 4 mg, and Tyramine, 140 mg	57 40	52 52
Van Bever et al. [13]; 1989	Single-arm pre–post intervention	Belgium	Elemental diet then DBPCC, both administered via nasogastric tube	6	NR	(5 mo–13.8 yo)	0–4 point scale * Physician assessment	Tyramine, 20 mg Acetylsalicylic acid, 100 mg	1 4	6 6
Worm et al. [10]; 2009	Non-randomised pre–post intervention	Germany	Elimination diet then DBPCC; healthy controls	36	8	32 yo (+/−1.4 y)	SCORAD	Histamine hydrochloride, 0.75 mg/kg and 1.5 mg/kg body weight, capsule	11	12

Abbreviations: AD, atopic dermatitis; yo, years old; mo, months; n, number of participants; NR, not reported; DBPCC, double-blind placebo-controlled challenge; RCT, randomised controlled trial; SCORAD, Scoring Atopic Dermatitis. * 0–4 point scale: No details were provided regarding the scale; a standard sheet was used to score DBPCC before and during the first 4 h after provocation. All positive reactions occurred within 10 min, with no late reactions. Nil placebo reactions.

**Table 2:** Overview of prevalence of pharmacological food intolerance.

Food Intolerance by Type	Challenge	Prevalence	Confidence Intervals	Heterogeneity	Certainty of Evidence
Histamine intolerance	Elimination diet	41%	95% CI, 25–58%	I^2^ = 52.78%	Low
Histamine intolerance	Oral challenge	31%	95% CI, 20–41%	I^2^ = 0.01%	Low
Amine intolerance	Oral challenge	32%	95% CI, 16–48%	I^2^ = 34.91%	Low
Salicylate intolerance	Oral challenge	53%	95% CI, 44–62%	I^2^ = 0.00%	Low

## Supplementary Materials

The following supporting information can be downloaded at: https://www.mdpi.com/article/10.3390/nu17101628/s1, Table S1. Joanna Briggs Institute Checklist for Prevalence Studies; Table S2. Characteristics of Excluded Studies Investigating the Prevalence of Chocolate Intolerance on Atopic Dermatitis; Table S3. Characteristics of Excluded Studies Investigating the Prevalence of Coffee Intolerance in Atopic Dermatitis; Table S4. GRADE Approach Assessment; Figure S1. Joanna Briggs Institute Checklist for Prevalence Studies Quality Assessment.

## Abbreviations

AD: atopic dermatitis; DBPCC, double-blind placebo-controlled challenge; JBI, Joanna Briggs Institute.
