# Supplementary material for: Prevalence of Intolerance to Amines and Salicylates in Individuals with Atopic Dermatitis: A Systematic Review and Meta-Analysis
Source: Nutrients. 2025 May 9;17(10):1628. doi: 10.3390/nu17101628 (PMC12114600; doi:10.3390/nu17101628)
Supplement: Supplementary file 1 [file nutrients-17-01628-s001.zip › nutrients-3603422-supplementary.pdf]

Supplement

Prevalence of Intolerance to Amines and Salicylates in Individuals with Atopic Dermatitis: A Systematic Review and Meta-Analysis

Contents

Methods 2

Search Strategy 2

Tables 8

Table S1. Joanna Briggs Institute Checklist for Prevalence Studies 8

Table S2. Characteristics of Excluded Studies Investigating the Prevalence of Chocolate Intolerance on Atopic Dermatitis 11

Table S3. Characteristics of Excluded Studies Investigating the Prevalence of Coffee Intolerance in Atopic Dermatitis 12

Table 4. GRADE Approach Assessment 13

Figures 14

Figure S1. Joanna Briggs Institute Checklist for Prevalence Studies Quality Assessment .. 14

Methods

Search Strategy

1. PubMed / MEDLINE

Search conducted (in incognito window) on 20/04/2023, yielding 344 results, and again on 22/04/2024, with filter “1 year”, yielding 21 results.

("atopic dermatitis"[Title/Abstract] OR "dermatitis, atopic"[MeSH Terms] OR "eczema"[MeSH Terms] OR "eczema"[Title/Abstract] OR "Neurodermatitis"[Title/Abstract] OR "Neurodermatitis"[MeSH Terms] OR "Besnier's prurigo"[Title/Abstract] OR "eczematous"[Title/Abstract] OR ("besnier*"[Title/Abstract] AND "prurigo"[Title/Abstract]))

AND

("food intolerance"[MeSH Terms] OR "food intolerance*"[Title/Abstract] OR "food chemical intolerance*"[Title/Abstract] OR "food sensitivity"[Title/Abstract] OR "food sensitivities"[Title/Abstract] OR "pseudoallergen"[Title/Abstract] OR "pseudo-allergen"[Title/Abstract] OR "pseudoallergic"[Title/Abstract] OR "histamine intolerance"[Title/Abstract] OR "amine intolerance"[Title/Abstract] OR "tyramine"[Title/Abstract] OR "phenylethylamine"[Title/Abstract] OR "tryptamine"[Title/Abstract] OR "vasoactive amines"[Title/Abstract] OR "ingested histamine"[Title/Abstract] OR "histamine sensitivity"[Title/Abstract] OR "salicylate*"[Title/Abstract] OR "salicylic acid"[Title/Abstract] OR "acetylsalicylic acid"[Title/Abstract] OR "willow bark"[Title/Abstract] OR "aspirin"[Title/Abstract] OR "low-histamine diet"[Title/Abstract] OR "histamine-free diet"[Title/Abstract] OR

"caffeine"[Title/Abstract] OR "caffeinated"[Title/Abstract] OR "coffee"[Title/Abstract] OR "chocolate*"[Title/Abstract] OR "theobromine"[Title/Abstract])

2. EMBASE (Elsevier)

Search conducted (in incognito window) on 20/04/2023, yielding 1505 results, and again on 22/04/2024, with publication years filter "2023-2024", yielding 145 results.

('atopic dermatitis':ti,ab OR 'dermatitis, atopic'/exp OR eczema/exp OR eczema:ti,ab OR Neurodermatitis:ti,ab OR Neurodermatitis/exp OR 'Besnier* prurigo':ti,ab OR eczematous:ti,ab OR (besnier*:ti,ab AND prurigo:ti,ab))
AND ('nutritional intolerance'/exp OR 'food intolerance*':ti,ab OR 'food chemical intolerance*':ti,ab OR 'food sensitivity':ti,ab OR 'food sensitivities':ti,ab OR pseudoallergen:ti,ab OR pseudo-allergen:ti,ab OR pseudoallergic:ti,ab OR 'histamine intolerance':ti,ab OR 'amine intolerance':ti,ab OR tyramine:ti,ab OR phenylethylamine:ti,ab OR tryptamine:ti,ab OR 'vasoactive amines':ti,ab OR 'ingested histamine':ti,ab OR 'histamine sensitivity':ti,ab OR salicylate*:ti,ab OR 'salicylic acid':ti,ab OR 'acetylsalicylic acid':ti,ab OR 'willow bark':ti,ab OR aspirin:ti,ab OR 'low-histamine diet':ti,ab OR 'histamine-free diet':ti,ab OR caffeine:ti,ab OR caffeinated:ti,ab OR coffee:ti,ab OR chocolate*:ti,ab OR theobromine:ti,ab)

3. CINAHL (EBSCO)

Search conducted (in incognito window) on 20/04/2023, yielding 59 results, and again on 22/04/2024, with publication years filter "2023-2024", yielding 3 results.

((TI "atopic dermatitis" OR AB "atopic dermatitis") OR (MH "dermatitis, atopic+") OR (MH eczema+) OR (TI eczema OR AB eczema) OR (TI Neurodermatitis OR AB Neurodermatitis) OR (MH Neurodermatitis+) OR (TI "Besnier's prurigo" OR AB "Besnier's prurigo") OR (TI eczematous OR AB eczematous) OR ((TI besnier* OR AB besnier*) AND (TI prurigo OR AB prurigo)))

AND

((MH "food intolerance+") OR (TI "food intolerance*" OR AB "food intolerance*") OR (TI "food chemical intolerance*" OR AB "food chemical intolerance*") OR (TI "food sensitivity" OR AB "food sensitivity") OR (TI "food sensitivities" OR AB "food sensitivities") OR (TI pseudoallergen OR AB pseudoallergen) OR (TI pseudo-allergen OR AB pseudo-allergen) OR (TI pseudoallergic OR AB pseudoallergic) OR (TI "histamine intolerance" OR AB "histamine intolerance") OR (TI "amine intolerance" OR AB "amine intolerance") OR (TI tyramine OR AB tyramine) OR (TI phenylethylamine OR AB phenylethylamine) OR (TI tryptamine OR AB tryptamine) OR (TI "vasoactive amines" OR AB "vasoactive amines") OR (TI "ingested histamine" OR AB "ingested histamine") OR (TI "histamine sensitivity" OR AB "histamine sensitivity") OR (TI salicylate* OR AB salicylate*) OR (TI "salicylic acid" OR AB "salicylic acid") OR (TI "acetylsalicylic acid" OR AB "acetylsalicylic acid") OR (TI "willow bark" OR AB "willow bark") OR (TI aspirin OR AB aspirin) OR (TI "low-histamine diet" OR AB "low-histamine diet") OR (TI "histamine-free diet" OR AB "histamine-free diet") OR (TI caffeine OR AB caffeine) OR (TI caffeinated OR AB caffeinated) OR (TI coffee OR AB coffee) OR (TI chocolate* OR AB chocolate*) OR (TI theobromine OR AB theobromine))

4. COCHRANE CENTRAL

Search conducted on 20/04/2023, yielding 56 trial results.

("atopic dermatitis":ti,ab OR [mh "dermatitis, atopic"] OR [mh eczema] OR eczema:ti,ab OR Neurodermatitis:ti,ab OR [mh Neurodermatitis] OR "Besnier's prurigo":ti,ab OR eczematous:ti,ab OR (besnier*:ti,ab AND prurigo:ti,ab))

AND

([mh "food intolerance"] OR ("food" NEXT intolerance*):ti,ab OR ("food chemical" NEXT intolerance*):ti,ab OR "food sensitivity":ti,ab OR "food sensitivities":ti,ab OR pseudoallergen:ti,ab OR pseudo-allergen:ti,ab OR pseudoallergic:ti,ab OR "histamine intolerance":ti,ab OR "amine intolerance":ti,ab OR tyramine:ti,ab OR phenylethylamine:ti,ab OR tryptamine:ti,ab OR "vasoactive amines":ti,ab OR "ingested histamine":ti,ab OR "histamine sensitivity":ti,ab OR salicylate*:ti,ab OR "salicylic acid":ti,ab OR "acetylsalicylic acid":ti,ab OR "willow bark":ti,ab OR aspirin:ti,ab OR "low-histamine diet":ti,ab OR "histamine-free diet":ti,ab OR caffeine:ti,ab OR caffeinated:ti,ab OR coffee:ti,ab OR chocolate*:ti,ab OR theobromine:ti,ab)

Grey Literature Searches

1. GOOGLE

Search conducted (in incognito window) on 27/04/2023, yielding 573 results.

("food intolerance" OR "salicylate sensitivity" OR "salicylate intolerance" OR "histamine intolerance" OR "amine intolerance") site:gov.au filetype:pdf

Search conducted (in incognito window) on 27/4/2023, yielding 123 results.

("atopic dermatitis" OR "eczema" OR "Neurodermatitis" OR "Besnier's prurigo") AND ("food chemical intolerance*" OR "food sensitivity" OR "food sensitivities" OR "pseudoallergen" OR "pseudo-allergen" OR "pseudoallergic" OR "histamine intolerance" OR "amine intolerance") site:gov filetype:pdf -nih.gov

2. PROQUEST

Search conducted (in incognito window) on 27/04/2023, yielding 5 results.

LIMITED TO: anywhere except full text – NOFT

noft(("atopic dermatitis" OR "eczema" OR "Neurodermatitis" OR "Besnier's prurigo") AND ("food intolerance" OR "food chemical intolerance*" OR "food sensitivity" OR "food sensitivities" OR "pseudoallergen" OR "pseudo-allergen" OR "pseudoallergic" OR "histamine intolerance" OR "amine intolerance" OR "tyramine" OR "phenylethylamine" OR "tryptamine" OR "vasoactive amines" OR "ingested histamine" OR "histamine sensitivity" OR "salicylates" OR "salicylic acid" OR "acetylsalicylic acid" OR "low-histamine diet" OR "histamine-free diet" OR "caffeine" OR "caffeinated" OR "coffee" OR "chocolate" OR "theobromine"))

OR

("atopic dermatitis" OR "eczema" OR "Neurodermatitis" OR "Besnier's prurigo") AND ("food intolerance" OR "food chemical intolerance*" OR "food sensitivity" OR "food sensitivities" OR "pseudoallergen" OR "pseudo-allergen" OR "pseudoallergic" OR "histamine intolerance" OR "amine intolerance" OR "tyramine" OR "phenylethylamine" OR "tryptamine" OR "vasoactive amines" OR "ingested histamine" OR "histamine sensitivity" OR "salicylates" OR "salicylic acid" OR

"acetylsalicylic acid" OR "low-histamine diet" OR "histamine-free diet" OR "caffeine" OR "caffeinated" OR "coffee" OR "chocolate" OR "theobromine")

3. Open Access Theses and Dissertations (OATD)

Search conducted (in incognito window) on 27/04/2023.

"atopic dermatitis" OR "eczema" AND ("histamine intolerance" OR "salicylate intolerance" OR "food intolerance" OR “food chemical intolerance*”)

Supplementary Tables

Table S1. Joanna Briggs Institute Checklist for Prevalence Studies [1]

First author and year	Chemical / food subgroup,	Was the sample frame appropriate to address the target population? Yes/No/Unclear	Were study participants sampled in an appropriate way?	Were the study subjects and setting described in detail? Yes/No/ Unclear	Were valid methods used for the identification of the condition? Yes/No/Unclear (method)	Was the condition measured in standard, reliable way for all participants? Yes/No/Unclear (method)	Was the response rate adequate, and if not, was the low response rate managed appropriately?	Overall appraisal: Include/ exclude
Worm, [2] 2009	Histamine	Y	Y	Y	Y (Hanifin & Rajka and SCORAD)	Y (DBPCC)	Unclear No information	Include
Fiedler, [3] 2005	Histamine	Unclear	Unclear	N	Y SCORAD	Y (DBPCC)	Unclear No information	Include
Wantke, [4] 1993	Histamine	Unclear	Unclear	N	Unclear	N (Reduction in symptoms or medications by ≥50%)	Unclear No information	Exclude (high risk)
Maintz, [5] 2006	Low histamine diet	N	Y	N	Y (Hanifin & Rajka, Bos, SCORAD)	Y (SCORAD)	Y	Exclude (wrong outcome)
Loblay, [6] 1986	Acetyl salicylic acid; Tyramine; phenylethyla mine	Y	Unclear	N	Unclear	Y (DBPCC)	Unclear No information	Include (poorly reported)
Van Bever, [7] 1989	Acetyl salicylic acid; Tyramine	Y	Y	Y	Y (Hanifin and Lobitz)	Y (DBPCC)	Y (1 child lost to follow up)	Include

Steinman, [8] 1994	Salicylate Chocolate Cocoa Coffee	Unclear	Unclear	N	Unclear	N (self-report)	Unclear No information	Exclude (critical risk; wrong outcome)
Greenlees, [9] 1998	Salicylate, amines	Unclear	Unclear	N	N relied on medical records from Dr. Soutter	N (used different methods including self-reports, medical and dietetic records)	N/A (data were partly retrospective)	Exclude (critical risk of bias, self-reports)
Pike, [10] 1989	Chocolate	Unclear	Unclear	N	N (Atherton method was modified)	N AD severity measured by one clinician (lead author). Modified visual score system by Atherton was used (Atherton is one of the authors)	Y (65 out of 66 completed the diet)	Exclude (high risk; wrong outcome)
Čelakovská, [11] 2014	Cocoa Chocolate	Unclear	Unclear	N	Y (SCORAD; Hanifin and Rajka)	N (self-report; could be any symptomatic reaction, not exclusively AD)	N/A (data were retrospective)	Exclude (critical risk of bias; wrong outcome)
Uenishi, [12] 2008	Chocolate	Unclear	No information	N	Y (Hanifin and Rajka)	Y (open challenge test in hospital setting up to 48-h later)	Unclear/ No information	Exclude (critical risk of bias)
Uenishi, [13] 2004	Chocolate	Unclear	No information	N	Unclear	No information	Unclear/ No information	Exclude (critical risk of bias)
Uenishi, [14] 2003	Chocolate Coffee	Unclear	No information	N	Y (Hanifin and Rajka)	Y (open challenge test in hospital	Unclear/ No information	Exclude (critical risk of bias)

						setting up to 48-h later)		
Veien, [15] 1987	Coffee	N (all subjects had dermatitis, eczema or urticaria caused by excessive coffee consumption: >10 cups/day)	No information	N	N/Unclear (DBPCC had missing data, including outcome scoring method)	N (self-reports; and DBPCC in subgroup; DBPCC had missing data, including scoring method)	Unclear	Exclude (critical risk of bias: missing data and population not representative of AD)

Table S2. Characteristics of Excluded Studies Investigating the Prevalence of Chocolate Intolerance on Atopic Dermatitis

Source (first author, date)	Study Design	Country	Intervention	n, Total	n, Male	Age, Mean (SD), Range*	Scoring Method	Food	n, Oral Challenge Responders	Prevalence
Pike, [10] 1989	Single arm pre-post intervention	United Kingdom	Elimination diet then oral challenge	5	NR	0.6-16.8 yo	Method by Atherton, et al. 1978	Chocolate	2	40%
Uenishi, [14] 2003	Single arm pre-post intervention	Japan	Elimination diet then open food challenge	195	81	23<br><br>16-53 yo	5-point scale	Chocolate	34	17.44%
Uenishi, [13] 2004	Single arm pre-post intervention	Japan	Elimination diet then open food challenge	59	28	8<br><br>3 – 12 yo	4-point scale	Chocolate	28	47.46%
Uenishi, [12] 2008	Single arm pre-post intervention	Japan	Elimination diet then open food challenge	69	NR	3 to 15 yo	5-point scale	Chocolate	24	34.78%

*Age shown in years, mean and (SD), where given. Abbreviations: yo, years old; mo, months; n, number of participants; NR, not reported.

The studies by Čelakovská [11] and Steinman [8] were excluded from the table because the self-reports did not specify whether adverse reactions to chocolate, cacao or cocoa resulted in the aggravation of atopic dermatitis, as they may have involved other symptoms, making the outcome potentially irrelevant.

Table S3. Characteristics of Excluded Studies Investigating the Prevalence of Coffee Intolerance in Atopic Dermatitis

Source (first author, date)	Study Design	Country	Intervention	n, Total	n, Male	Age, Mean (SD), Range	Scoring Method	Chemical/ Beverage	n, Oral Challenge Responders	Prevalence
Uenishi, [14] 2003	Single arm<br>pre-post<br>intervention	Japan	Elimination diet then open food challenge	195	81	23<br>16-53 yo	5-point scale	Coffee	18	9.23%
Veien, [15] 1987	Self-reports	Denmark	Questionnaire, sent 6mo to 5y after diagnosis	21	5	37<br>15 to 81 yo	Self-reported	Coffee	1<br>(<1 cup/day; flares reported in all 21 subjects after resuming excess coffee intake)	4.76%
	DBPCC		DBPCC	5	NR	NR	NR	Caffeine	0	0%

*Age shown in years, mean and (SD), where given. Abbreviations: yo, years old; mo, months; y, years; n, number of participants; NR, not reported.

The Steinman study [8] was excluded from the table because the self-reports did not specify whether adverse reactions to coffee resulted in the aggravation of atopic dermatitis or other symptoms, making the outcome potentially irrelevant.

Table S4. GRADE Approach Assessment

**Chemical subgroup and challenge**	**Figure**	**Hetero-geneity**	**Can heterogeneity Inconsistencies be explained Y/N**	**Do confidence intervals overlap adequately? Y/N**	**Risk of bias, overall body of evidence clinically meaningful? Y /N**	**Downgrade evidence? Y/N**
HISTAMINE, DBPCC	Figure 2	0.01%	n/a	Y	Y	Y
HISTAMINE, LOW HISTAMINE DIET	Figure 3	52.78%	Yes, Worm et al.[2] excluded extra chemicals including salicylates	Y	Y Result (31%) is similar to amine challenge result (32%)	Y
SALICYLATES, DBPCC	Figure 2	0.00%	n/a	Y	Y May indicate aspirin intolerance	Y
AMINES, DBPCC	Figure 2	34.91%	Yes, Loblay and Swain[6] used two types of amines which elicited a higher result, and Van Bever et al.[7] used one	Y	Y	Y

Abbreviations: DBPCC, double-blind placebo-controlled challenge

Supplementary Figures

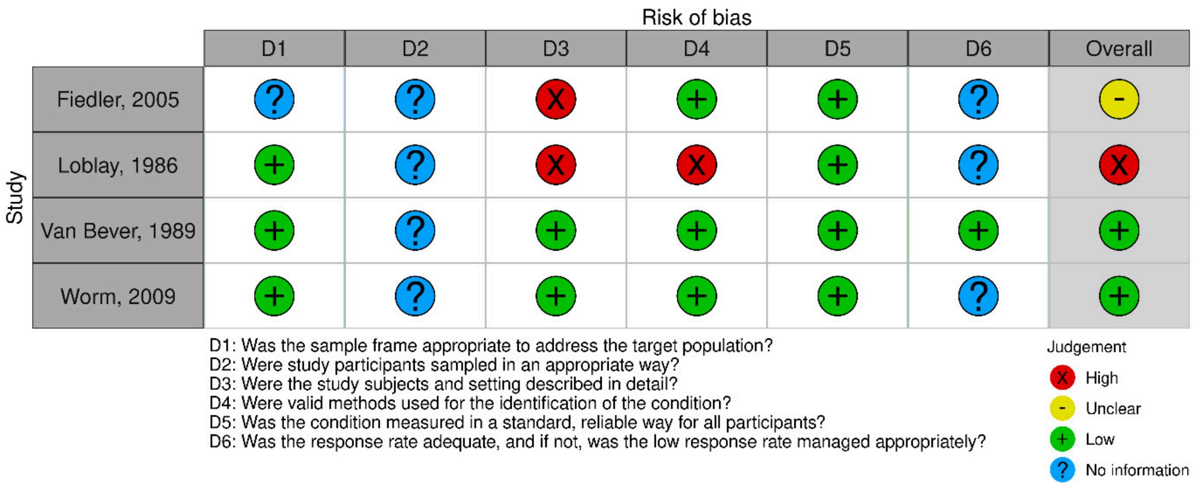

Figure S1. Joanna Briggs Institute Checklist for Prevalence Studies Quality Assessment [1, 16]

References

1. Munn, Z., Moola, S., Lisy, K., Riitano, D., and Tufanaru, C., *Chapter 5: Systematic reviews of prevalence and incidence*. JBI Manual for Evidence Synthesis. , ed. M.Z. Aromataris E. 2020: Joanna Briggs Institute.
2. Worm, M., Fiedler, E.M., Dolle, S., Schink, T., Hemmer, W., Jarisch, R., and Zuberbier, T., *Exogenous histamine aggravates eczema in a subgroup of patients with atopic dermatitis.* Acta Derm Venereol, 2009. **89**(1): p. 52-6.
3. Fiedler, E.M., Forschner, K., Focke, M., Hemmer, W., Jarisch, R., Zuberbier, T., and Worm, M., *Nutrition and eczema diseases - Importance of exogenously supplied histamine in patients with atopic dermatitis.* Dermatology in Work and the Environment 2005. **53**: p. 93-96.
4. Wantke, F., Gotz, M., and Jarisch, R., *[The histamine-free diet].* Der Hautarzt, 1993. **44**(8): p. 512-6.
5. Maintz, L., Benfadal, S., Allam, J.P., Hagemann, T., Fimmers, R., and Novak, N., *Evidence for a reduced histamine degradation capacity in a subgroup of patients with atopic eczema.* J Allergy Clin Immunol, 2006. **117**(5): p. 1106-12.
6. Loblay, R.H. and Swain, A.R., *Food Intolerance.* Recent Advances in Clinical Nutrition, 1986. **2**: p. 169-177.
7. Van Bever, H.P., Docx, M., and Stevens, W.J., *Food and food additives in severe atopic dermatitis.* Allergy, 1989. **44**(8): p. 588-94.
8. Steinman, H.A. and Potter, P.C., *The precipitation of symptoms by common foods in children with atopic dermatitis.* Allergy Proc., 1994. **15**(4): p. 203-10.
9. Greenlees, N., *Food intolerance in children with eczema*. Thesis, University of Sydney, 1998.
10. Pike, M.G., Carter, C.M., Boulton, P., Turner, M.W., Soothill, J.F., and Atherton, D.J., *Few food diets in the treatment of atopic eczema.* Arch Dis Child, 1989. **64**(12): p. 1691-8.
11. Čelakovská, J., Ettler, K., Ettlerova, K., and Vaneckova, J., *Food hypersensitivity in patients over 14 years of age suffering from atopic dermatitis.* Indian J Dermatol, 2014. **59**(3): p. 316.
12. Uenishi, T., Sugiura, H., Tanaka, T., and Uehara, M., *Role of foods in irregular aggravation of skin lesions in children with atopic dermatitis.* J Dermatol, 2008. **35**(7): p. 407-12.
13. Uenishi, T., Sugiura, H., and Uehara, M., *Involvement of food in irregular exacerbations of childhood atopic dermatitis.* Skin Science, 2004. **3**: p. 93-96.
14. Uenishi, T., Sugiura, H., and Uehara, M., *Role of foods in irregular aggravation of atopic dermatitis.* J Dermatol, 2003. **30**(2): p. 91-7.
15. Veien, N.K., Hattel, T., Justesen, O., Norholm, A., *Dermatoses in coffee drinkers.* The Dermatology Clinic, 1987. **40**: p. 421-422.
16. McGuinness, L.A. and Higgins, J.P.T., *Risk-of-bias VISualization (robvis): An R package and Shiny web app for visualizing risk-of-bias assessments.* Res Synth Methods, 2021. **12**(1): p. 55-61.
